# Nonalcoholic Fatty Liver Disease, Diabetes Mellitus and Cardiovascular Disease: Newer Data

**DOI:** 10.1155/2013/450639

**Published:** 2013-04-03

**Authors:** A. N. Mavrogiannaki, I. N. Migdalis

**Affiliations:** 2nd Medical Department and Diabetes Center, NIMTS Hospital, 12 Monis Petraki, 11521 Athens, Greece

## Abstract

Nonalcoholic fatty liver disease (NAFLD) is the most common, chronic liver disease worldwide. Within this spectrum, steatosis alone is apparently benign, while nonalcoholic steatohepatitis may progress to cirrhosis and hepatocellular carcinoma. NAFLD is strongly associated with obesity, dyslipidemia, type 2 diabetes mellitus, and cardiovascular disease. The pathogenesis of hepatic steatosis is not clearly known, but its main characteristics are considered insulin resistance, mitochondrial dysfunction, increased free fatty acids reflux from adipose tissue to the liver, hepatocyte lipotoxicity, stimulation of chronic necroinflammation, and fibrogenic response. With recent advances in technology, advanced imaging techniques provide important information for diagnosis. There is a significant research effort in developing noninvasive monitoring of disease progression to fibrosis and response to therapy with potential novel biomarkers, in order to facilitate diagnosis for the detection of advanced cirrhosis and to minimize the need of liver biopsy. The identification of NAFLD should be sought as part of the routine assessment of type 2 diabetics, as sought the microvascular complications and cardiovascular disease, because it is essential for the early diagnosis and proper intervention. Diet, exercise training, and weight loss provide significant clinical benefits and must be considered of first line for treating NAFLD.

## 1. Introduction

Nonalcoholic fatty liver disease (NAFLD) includes a wide spectrum of liver clinicopathologic conditions, ranging from pure fatty steatosis (fatty infiltration in >5% of hepatocytes) which is apparently a benign condition to nonalcoholic steatohepatitis (NASH), which may progress to cirrhosis, liver failure, and hepatocellular carcinoma (HCC). It is characterized by excessive fat accumulation in the liver parenchyma of patients who have no history of alcohol abuse (<20 g per day in men and <10 g in women). NASH is characterized by biochemical evidence of hepatocellular damage (elevation of aminotransferase levels), histological findings of the type of alcoholic hepatitis (steatosis, lobular inflammatory cell infiltration, Mallory's hyaline, and fibrosis), and no other cause of liver damage.

NAFLD is the most common liver disease worldwide. Its reported prevalence varies, depending on the population of the study and using diagnostic criteria. In the general population exceeds 15%, but it is far greater in the overweight, obese, and in subjects with type 2 diabetes (T2DM) as well type 1 diabetics [1–3]. Recent data indicate high prevalence in adolescents. The overall prevalence of NAFLD is reported 12.5%, increasing to 23.0% in overweight/obese, higher in boys than in girls [4]. NAFLD is described in the 60% of the subjects with mixed hyperlipidemia, and in 83% of those with both mixed hyperlipidemia and an elevated serum alanine aminotransferase (ALT) [5]. 

NAFLD is strongly associated with T2DM and cardiovascular disease (CVD). It is characterized by insulin resistance and mitochondrial dysfunction [6]. Indeed, there is a gradual increase in the severity of insulin resistance in the range of NAFLD which may contribute to the evolution of liver damage. Also, is associated with an increased risk of kidney disease in subjects with multiple CVD risk factors and tends to be considered as an independent CVD marker [7]. Diabetes, dyslipidemia, hypertension and CVD coexist more frequently in individuals with NAFLD [8].

Health cost appears to be greater in NAFLD individuals than in general population. When were used data from the “Study of Health in Pomerania,” Germany, to assess the relation of fatty liver disease to self-reported health care utilization and costs at baseline and 5 years, in a general population cohort study of 4310 adults aged 20 to 79 years at baseline, subjects with NAFLD and increased serum ALT levels, after controlling for comorbid condition, had 26% higher overall health care costs [9].

## 2. NAFLD and T2DM

From various studies, the prevalence of NAFLD seems higher in type 2 diabetics than in general population, independent of glycemic control [10]. Type 2 diabetics have approximately 80% more fatty liver compared with nondiabetics matched for age and sex [11]. In a study of 2589 individuals from the community-based Framingham Heart Study, after multivariate adjustment for other fat depots (visceral adipose tissue, waist circumference, and body mass index (BMI)), fatty liver remained associated with diabetes, impaired fasting glucose, hypertension, metabolic syndrome, HDL cholesterol, triglycerides, and adiponectin levels (all *P* < 0.001), whereas associations with systolic (SBP) and diastolic (DBP) blood pressure were attenuated (*P* > 0.05) [12].

There are studies which highlight diabetes as risk marker for NAFLD/NASH appearance. In a study of 458 Italian patients with histological proven NASH, diabetes was the most significant marker of NASH and fibrosis and in those with normal ALT [13]. Severe fibrosis was independently predicted by diabetes (OR = 1.8; 95% CI, 1.4–2.3) in the overall series and in those with normal ALT and insulin resistance according to homeostasis model assessment (HOMA-IR) (OR = 1.97; 95% CI, 1.2–3.7) in patients with normal ALT [13]. In a cohort of 827 patients with NAFLD, advanced fibrosis was associated with insulin resistance [14]. 

Several studies have shown that NAFLD predicts the appearance of diabetes independently of conventional risk factors, as obesity, insulin resistance, and metabolic syndrome, suggesting that NAFLD could have a direct causal relationship with the appearance of diabetes, probably by promoting the insulin resistance [15]. It has been shown that increased liver enzymes predict T2DM independent of obesity [16]. In another study, metabolic changes (lipid, liver enzymes, blood pressure, and body weight) potentially associated with conversion to diabetes were investigated, it was found that in subjects who converted to new-onset diabetes, ALT (*P* = 0.0005) and triglycerides' (*P* = 0.030) concentrations are increased in absence of changes in body weight up to 18 months before the diabetes manifestation, but neither parameters increased significantly in nonconverters with high baseline glucose concentrations (>6.1 mmol/L) [17].

The poor controlled diabetes, also, promotes or worsens hepatic steatosis, thus feeding a vicious cycle that binds the two situations.

Therefore, hepatic steatosis, diabetes, and metabolic syndrome are part of the same disease process ultimately leading to increased cardiovascular morbidity and mortality risk. 

## 3. NAFLD and CVD

Several prospective, epidemiological studies have shown that elevation of liver enzymes and ultrasonographic appearance of hepatic steatosis are predictors of CVD independent of conventional risk factors [18, 19]. Indeed, among 1221 apparently healthy subjects who were recruited from a health check-up program, NAFLD was a predictor of CVD independent of conventional risk factors (odds ratio 4.12, 95% CI, 1.58 to 10.75, *P* = 0.004) and had central role in the cardiovascular risk of metabolic syndrome [19]. Metabolic syndrome was also independently associated with cardiovascular events, but simultaneous inclusion of NAFLD and metabolic syndrome in a multivariate model revealed that NAFLD but not metabolic syndrome retained a statistically significant correlation with CVD [19]. In a study of subjects with elevated ALT levels was found NAFLD and increased coronary heart disease (CHD), as assessed by Framingham risk score [20]. In another study, NAFLD subjects' survival was found lower compared with matched controls after a mean followup of 13.7 years [21]. Mortality was not increased in patients with steatosis, but it was found higher in NASH patients. These subjects more often died from cardiovascular (*P* = 0.04) and liver-related (*P* = 0.04) causes [21].

Subjects with NAFLD have significantly higher carotid artery intima-media thickness (IMT), a marker of subclinical atherosclerosis, comparing with those without fatty liver disease (mean IMT = 0.417 mm versus 0.395 mm, *P* < 0.001), impaired endothelial function, and lower concentrations of adiponectin [4, 22]. IMT is strongly associated with degree of hepatic steatosis, necroinflammation, and fibrosis among NAFLD patients (*P* < 0.001 for all) [23]. Similarly, the severity of histological features of NAFLD independently predicted carotid IMT (*P* < 0.001) after adjustment for all confounders associated with the presence and severity of coronary atherosclerosis and cardiovascular disease [23]. These results suggest that the severity of liver histopathology among NAFLD patients is strongly associated with early carotid atherosclerosis, independent of classical risk factors, insulin resistance, and the presence of metabolic syndrome [23]. When the vasodilatory response of the brachial artery was assessed in response to ischemia (a test of endothelial function) as well as cardiovascular profile (10-year risk of coronary events) in 52 NAFLD cases and 82 age- and sex-matched controls, were endothelial dysfunction and increased risk of cardiovascular events in NAFLD subjects compared with controls observed [24].

It seems that NASH can predict a more atherogenic risk profile in a manner that is partly independent of the contribution of visceral adiposity. In a study, the differential contribution of NASH and visceral adiposity to nontraditional cardiovascular risk biomarkers in adult men was assessed [25]. 45 consecutive, overweight male patients with biopsy-proven NASH, 45 overweight men without ultrasound-diagnosed hepatic steatosis and 45 healthy male volunteers were included. All participants were matched for age; NASH and overweight patients were also matched for BMI and visceral adiposity (as estimated by abdominal ultrasonography) [25]. Plasma concentrations of high-sensitivity C-reactive protein (hs-CRP), fibrinogen, and plasminogen activator inhibitor-1 (PAI-1) activity were found markedly lower in nonobese healthy volunteers, intermediate in overweight nonsteatotic patients, and the highest in overweight subjects with biopsy-proven NASH, after adjustment for age, BMI, smoking, plasma triglycerides, and insulin resistance [25]. Also, the highest concentrations of adiponectin were found in nonobese healthy subjects and the lower in those with biopsy-proven NASH [25]. 

The relationship between hypertension and NAFLD has also been investigated. Higher prevalence of NAFLD in nonobese nondiabetic hypertensive patients with normal liver enzymes compared with adjusted nonhypertensive subjects. It has been found [26].

Abnormal left ventricular energy metabolism, fat accumulated in the epicardial area despite normal left ventricular morphological features, and systolic and diastolic functions, in nondiabetic young with fatty liver compared with nondiabetic matched for anthropometric features without fatty liver. It has also been reported [27]. It is interesting that, the addition of pioglitazone to insulin therapy, in type 2 diabetics, reduced myocardial and hepatic steatosis [28].

NAFLD is significantly associated with an increased CVD risk (odds ratio (OR) 1.84, 95% CI, 1.4–2.1, *P* < 0.001) among type 2 diabetics independent of classical risk factors, liver enzymes, or metabolic syndrome [29]. The independent association among NAFLD and CVD is supported by another large study following type 2 diabetics for 6.5 years [30]. Significant association of NAFLD with incident CVD (hazart ratio 1.96 (1.4–2.7), *P* < 0.001) by adjustment for sex, age, smoking, diabetes duration, HbA1c, LDL-cholesterol and medications was found [30].

NAFLD in type 1 diabetics is associated with higher prevalence of CVD compared with non-NAFLD type 1 diabetics independently of age, sex, smoking, diabetes duration, HbA1c, LDL-cholesterol, HDL-cholesterol, triglycerides, SBP, and medication use (adjusted OR 7.36, 95% CI, 1.60–34.3, *P* < 0.01) [3].

## 4. Pathogenesis of NAFLD

The pathogenesis of hepatic steatosis remains poorly understood but its main characteristics are considered insulin resistance and mitochondrial dysfunction [6]. If insulin resistance precedes NAFLD or the opposite remains unanswered. The source of stored hepatic fat results from dietary carbohydrates and fatty acids and the release of fat from adipocytes, by lipolysis or de novo hepatic lipogenesis [6]. An imbalance between the mechanisms may modify the rate of fat oxidation and fat removal from the liver. Defects in multiple levels may tip the metabolic balance towards hepatic fat accumulation: excessive substrate supply to the liver (i.e., glucose and fatty acids), intrahepatic imbalance between lipid synthesis and oxidation; inadequate export to peripheral tissues; and a combination of the above [31]. Many molecular defects at these different steps have been described in NAFLD. 

### 4.1. Hepatic Insulin Resistance and NAFLD

Insulin resistance leads to hepatocyte steatosis by stimulation of insulin secretion and by increased lipolysis in adipose tissue, which increases circulating fatty acids. Increased uptake of circulating free fatty acids (FFA) by hepatocytes leads to mitochondrial beta-oxidation overload, with the consequent accumulation of fatty acids within hepatocytes. Fatty acids are substrates and inducers of the microsomal lipoxygenases cytochrome P-450 2E1 and 4A, resulting in the production of free oxygen radicals capable of inducing lipid peroxidation of hepatocyte membranes [32]. Hyperinsulinemia resulting from insulin resistance increases the synthesis of fatty acids in hepatocytes by increasing glycolysis and favors the accumulation of triglycerides within hepatocytes by decreasing hepatic production of apolipoprotein B-100. Reactive oxygen species (ROS) trigger lipid peroxidation, which causes cell death and releases malondialdehyde (MDA) and 4-hydroxynonena (HNE). MDA and HNE cause cell death; cross-link proteins, leading to the formation of Mallory's hyaline, and activate stellate cells promoting collagen synthesis. HNE has chemotactic activity for neutrophils, promoting tissue inflammation. ROS also induce the formation of cytokines tumor necrosis factor-*α*  (TNF-*α*), transforming growth factor  *β*  (TGF-*β*) and interleukin-8. TNF-*α*  and TGF-*β*  cause caspase activation and hepatocyte death. TGF-*β*  activates collagen synthesis by stellate cells and activates tissue transglutaminases, promoting the formation of Mallory's hyaline. The TNF-*α*  induced by ROS further impairs the flow of electrons along the respiratory chain in mitochondria. Mitochondrial ROS cause expression of the Fas ligand (a death receptor member of TNFR family) in hepatocytes, which normally express the membrane receptor Fas. The Fas ligand on one hepatocyte can interact with Fas on another hepatocyte, causing fractional killing [32]. 

Elevated levels of triacylglycerol (TAG), diacylglycerol (DAG) and free cholesterol, NF-*κ*B signaling activation, mitochondrial dysfunction, and FFA toxic role it have reported [6, 31]. 

### 4.2. Adipose Tissue Insulin Resistance and NAFLD

Adipose tissue insulin resistance is associated with increased hepatic fat synthesis, regardless of obesity [15]. Adipocytes account for approximately 60%–70% of the FFA used for hepatic triglyceride synthesis and very low-density lipoprotein (VLDL) secretion [31]. Many factors that regulate VLDL metabolism may promote steatosis. 

The adipocyte is a dynamic endocrine organ and nutrient sensor that tightly regulates energy supply. When nutrient supply exceeds adipose tissue adaptation, adipocyte hypertrophy and other poorly understood factors set off a pathologic adipocyte-macrophage crosstalk. The final result is adipocyte insulin resistance and the chronic release of FFA with toxic effects in distant tissues such as muscle, liver, and pancreatic  *β*-cells as well as on the heart and vascular bed [15]. The precise series of events occurring is incompletely understood. Although the mechanisms by which the hepatic fat aggregation is associated with adipose tissue inflammation were not elucidated, macrophages infiltration may contribute to adipose tissue insulin resistance.

Excessive FFA availability promotes the accumulation of intramyocellular lipids and the formation of a variety of fat-derived potentially toxic lipid metabolites such as ceramide and DAG that activate the IKK/NF-*κ*B pathway and cause insulin resistance. Inhibition of muscle insulin signaling and insulin resistance inhibits insulin action on adipose glucose uptake and lipid synthesis, further increasing the rate of FFA release into the circulation, and contributes to the development of insulin resistance and type 2 diabetes [15].

There is ample evidence that increases of plasma FFA concentrations cause hepatic insulin resistance, impair insulin signaling, and stimulate hepatic glucose production by driving both hepatic gluconeogenesis and glycogenolysis [15]. Induction of hepatic insulin resistance is following lipid infusion and correlates closely with increase in plasma FAA [15].

### 4.3. From Insulin Resistance to NASH

It is known that only 10%–25% of NAFLD subjects develop NASH [33]. Factors responsible for this evolution have been subject to extensive research but still remain incompletely understood. Still, data are fragmented and arise largely from rodents given the natural difficulties of assessing human liver tissue. One must keep in mind that there are significant metabolic/molecular differences between livers from humans and rodents and even between rodent species.

With these limitations in mind, in an effort to organize the current understanding of NAFLD and NASH, a framework is proposed on the progression from adipose tissue insulin resistance to NAFLD and NASH [31]. A prerequisite or “first step” for NASH appears to be adipose tissue insulin resistance, providing the necessary “lipotoxic environment” that ensures ample substrate supply to the liver (i.e., high FFA flux) and compensatory hyperinsulinemia that stimulates excessive hepatic triglyceride synthesis and the formation of toxic saturated fatty acids. The “second step” towards NASH is the development of hepatic steatosis and of a lipid pool from where lipid-derived toxic metabolites may activate inflammatory pathways. Dietary and genetic factors may condition the metabolic adaptation of the liver to this harmful environment. Compensated steatosis exacerbates hepatic insulin resistance, stimulates VLDL secretion, and increases mitochondrial beta-oxidation. If a new steady state is achieved, only benign steatosis and/or dyslipidemia (high triglyceride, low HDL-cholesterol) takes place. The “third step” for the progression from simple “bland” steatosis to active necroinflammation depends on the ability of the liver to adapt to longstanding triglyceride accumulation. If mitochondrial function cannot adapt to the increased FFA flux and respiratory oxidation collapses, lipid-derived toxic metabolites activate inflammatory pathways and hepatocyte lipotoxicity with stimulation of chronic necrosis and inflammation. Fibrosis is the final “fourth step,” involving chronic activation of hepatic stellate cells in a yet poorly understood cross-talk of Kupffer cells with hepatocytes. The magnitude of the crosstalk between hepatocytes, macrophages, and hepatic stellate cells determines the degree of the fibrogenic response and potential progression to cirrhosis. In this setting, low plasma adiponectin levels are believed to promote steatosis and fibrosis by allowing unchecked triglycerides synthesis and activation of hepatic stellate cells, respectively. 

The association between NAFLD, insulin resistance, and T2DM seems to be strong and partly due to genetic and environmental factors. The polymorphism of certain genes has been shown both in animal model and human studies that plays a significant role [34–36]. Diets rich in saturated fats, soft drinks, and meat and low in antioxidants, fish, and omega-3 fat are associated with development of NAFLD [37, 38]. 

## 5. Recent Advances in Diagnosis of NAFLD

The diagnosis of NAFLD/NASH is usually suspected in subjects with asymptomatic elevation of aminotransferase level, radiologic findings of fatty liver, or unexplained persistent hepatomegaly. The clinical diagnosis and liver tests have a poor predictive value with respect to histologic involvement. Imaging studies, although being of help in to determining the presence and amount of fatty infiltration of liver, cannot be used accurately determine the severity of liver damage. NAFLD is often diagnosed by a combination of clinical, laboratory and imaging data, but the clinical suspicion of NAFLD and its severity can only be confirmed with a liver biopsy [39]. Liver biopsy remains the best diagnostic tool for confirming NAFLD and evaluating necroinflammation/fibrosis, as well as the most sensitive and specific means of providing important prognostic information. Although liver biopsy is a relatively safe procedure when, performed by experienced clinicians, it has poor patient acceptance, it is not risk free and is difficult to be repeated.

Reliable and reproducible noninvasive methods for evaluating hepatocellular fat accumulation as well as the variable degree of hepatocyte necroinflammation (activity or grade of disease) and fibrosis (stage of disease), for frequent monitoring of disease progression, of treatment efficacy, and for prognosis assessment are strongly needed. With recent advances in technology, advanced imaging techniques (sonographic and magnetic elastography, magnetic spectroscopy) provide important information for diagnosis and usually diagnosis is based on these [40]. Several laboratory investigators try to identify potential novel biomarkers based on the current knowledge of the pathophysiologic mechanisms involved in NAFLD progression [41–43]. An ideal biomarker should be simple, reproducible, inexpensive, readily available, and accurate for a particular disease process. Potential rational targets for biomarkers development in NAFLD/NASH are based on the central role of inflammatory cytokines in the development of NAFLD, on the different oxidation products of several oxidation pathways, on mediators of fibrogenesis/fibrosis, on mediators/receptors involved in the hepatocyte apoptosis, and on breath biomarkers. Different mechanisms have been proposed including an increased production of reactive oxygen species and mitochondrial outer permeabilization, resulting in a cascade of events leading to inflammation (TNF-*α*, adiponectin, C-reactive protein, IL-6, Resistin, and visfatin), hepatocellular apoptosis (Fas, circulating active caspase 3), fibrogenesis, and fibrosis (TGF-*β*, tissue elasticity) [41]. It has evaluated the use of breath biomarkers in the study of NAFLD, such breath ethanol, ethane, and breath acetone [42]. Also, efforts attempts are made to identify noninvasive indicators of liver fibrosis by using routinely determined and easily available clinical and biochemical variables [44]. All these markers are under investigation and their clinical utility remains to be determined [41–44].

It is noted that with all above, diagnosis of significant fibrosis may be satisfactory, but that of steatosis and NASH without fibrosis is problematic.

## 6. Therapy of NAFLD

There is currently no established treatment for NAFLD or NASH, although weight loss is recommended [39]. Several pharmaceutical interventions have been evaluated but none has been approved for general use. Most treatment studies have focused on subjects with NASH because of their potential to progress to fibrosis and cirrhosis; however, the findings have been limited by variations in treatment endpoints and a paucity of randomized, placebo-controlled, powerful and of sufficient duration trials.

Lifestyle changes, mostly focused on weight loss, have been demonstrated to improve liver aminotransferases and histological findings in obese with fatty liver [45]. In overweight or obese individuals with biopsy-proven NASH, weight reduction achieved through lifestyle intervention leads to improvements in liver histology [46]. The content of diet slimming is of no particular importance (advising to avoid alcohol), if lead to weight loss. However, although weight loss appears to be beneficial, rapid weight loss after gastroplasty has been associated with increased hepatitis despite reductions in steatosis on liver biopsy [47]. Weight loss agents had no significant effects compared with weight loss only [48]. In obese patients undergoing bariatric surgery, hepatic steatosis is decreased from 53% to 32%, three months after surgery as overall mortality [49, 50]. Cross-sectional investigations have shown an independent association between physical fitness and hepatic triglyceride concentration [51]. Regular exercise reduces hepatic and visceral lipids in previously sedentary obese individuals even in absence of weight loss [52]. 

Several pharmaceutical interventions have attempted to NAFLD/NASH, with limited benefit overall. Some studies have been performed with drugs acting cytoprotective or antioxidants or tumor necrosis factor antagonists or decreasing cytokines production, inhibitors of TGF-*β*, and semisynthetic agonists of receptor Farsenoid X with unsuccessful or moderate results [53–58]. In recent years, drugs that inhibit the system renin-angiotensin and  *α*-receptors with moderate biochemical and histological response are studied [59]. Sought treatment options for NASH, especially when is accompanied by fibrosis, but there are not large randomized trials.

Administration of lipid-lowering drugs has been evaluated in patients with NAFLD/NASH, but not in large prospective controlled trials and it has been associated with biochemical and histological improvement, but not all studies. The use of statins appears to be safe in patients monitored closely, to treat hyperlipidemia [39, 60]. The long-term ezetimibe therapy can lead to improvement in metabolic, biochemical, and histological abnormalities of NAFLD [61]. In small uncontrolled studies moderate or no benefit from use of fibrates has reported [62, 63]. 

The common metabolic disorders of T2DM and NAFLD can explain the greater success of drugs used to treat diabetes, with the most studies to target the use of drugs that improve insulin resistance. Use of metformin in NAFLD patients does not appear to help and is not recommended in nondiabetic NAFLD/NASH patients [39, 58]. Clinical trials of pioglitazone have shown promising results (partial biochemical and histological efficacy) but the short-term effect and side effects may limit widespread acceptance [56]. At present, they are used only in clinical trials, while they can be used in type 2 diabetic patients with NAFLD/NASH. Intensive insulin therapy in type 2 diabetic patients with NAFLD/NASH appears to significantly decrease on steatosis [64]. GLP-1 receptors were detected on human hepatocytes and treatment with exendin-4 quantitatively reduced triglyceride stores compared with control-treated cells [65]. The current preclinical evidence shows that GLP-1 analogs and DPP-4 inhibitors can improve hepatic steatosis independent of weight loss but is controversial whether the pancreatic-type GLP-1 receptor is present or responsible for conferring the GLP-1 signal in the hepatocyte [66]. 

In patients with NAFLD/NASH, all cardiovascular risk factors (obesity, hypertension, hyperglycemia, and hyperlipidemia) are treated [39, 67]. The identification of NAFLD should be sought as part of assessment of diabetic patients for proper implementation of lifestyle and pharmaceutical interventions [68].

Liver transplantation is recommended in patients with decompensate cirrhosis due NASH as long as underlying comorbidities permit [32]. 

## 7. Natural History: Prognosis

NAFLD often follows a benign course but may leads to fibrosis, chirrosis due NASH and HCC. The evolution of fibrosis in NASH has been found in 25%–33% of the cases [69]. Factors favoring the evolution to cirrhosis are fibrosis, obesity (visceral), diabetes, and hypertension [70]. There is epidemiological evidence that NASH and cirrhosis are associated with increased HCC risk [71]. Mild steatosis is not associated with an increased risk mortality compared with general population [72]. In type 2 diabetics, presence of NAFLD is associated with increased total mortality, regardless of classic risk factors [73]. Diabetics with NAFLD have twice risk mortality compared with nondiabetics without NAFLD, with more common causes of death malignancy (33% of death) and liver related complications (19% of death) [73]. 

## 8. Conclusions

NAFLD is strongly associated with T2DM and CVD. Within this spectrum, steatosis alone is apparently benign, while nonalcoholic steatohepatitis may progress to cirrhosis and hepatocellular carcinoma. Its pathogenesis is complex and involves insulin resistance and mitochondrial dysfunction with increased FFA reflux from adipose tissue to the liver which play a key role in the chronic activation of inflammatory pathways and hepatocyte lipotoxicity with stimulation of chronic inflammation and necrosis. There is significant research effort in developing noninvasive monitoring of disease progression to fibrosis and response to therapy with potential novel biomarkers, which promise to facilitate diagnosis for the detection of advanced cirrhosis in order to minimize the need of liver biopsy, which are not used in clinical practice. The identification of NAFLD should be sought as part of routine assessment of diabetic patients, as sought the microvascular complications and CVD, because it is essential for the early diagnosis and proper implementation of lifestyle and pharmaceutical interventions. Diet, exercise, and weight loss provide significant clinical benefits and must be considered of first line for treating NAFLD/NASH.
